# The behavioral responses of a nocturnal burrowing marsupial (*Lasiorhinus latifrons*) to drone flight

**DOI:** 10.1002/ece3.7981

**Published:** 2021-08-01

**Authors:** Taylor Headland, Bertram Ostendorf, David Taggart

**Affiliations:** ^1^ School of Biological Science The University of Adelaide Adelaide SA Australia; ^2^ College of Science and Engineering Flinders University Bedford Park SA Australia; ^3^ School of Animal and Veterinary Science The University of Adelaide Urrbrae SA Australia

**Keywords:** anthropogenic impacts, drones, nocturnal observation, UAVs, vigilance behavior

## Abstract

The use of drones in wildlife research and management is increasing. Recent evidence has demonstrated the impact of drones on animal behavior, but the response of nocturnal animals to drone flight remains unknown. Utilizing a lightweight commercial drone, the behavioral response of southern hairy‐nosed wombats *(Lasiorhinus latifrons)* to drone flights was observed at Kooloola Station, Swan Reach, South Australia. All wombats flown over during both day and night flights responded behaviorally to the presence of drones. The response differed based on time of day. The most common night‐time behavior elicited by drone flight was retreat, compared to stationary alertness behavior observed for daytime drone flights. The behavioral response of the wombats increased as flight altitude decreased. The marked difference of behavior between day and night indicates that this has implications for studies using drones. The behavior observed during flights was altered due to the presence of the drone, and therefore, shrewd study design is important (i.e., acclimation period to drone flight). Considering the sensory adaptations of the target species and how this may impact its behavioral response when flying at night is essential.

## Graphical abstract

This study explores the disturbance behaviors of the southern hairy‐nosed wombat (*Lasiorhinus latifrons*) to daytime and night‐time drone flight. A lightweight UAV was flown over the wombats at 3 different altitudes to document vigilance behaviors. Wombats were found to respond behaviorally to the presence of the drone for all flight altitudes, regardless of time of day, and the behavioral response of the wombats intensified as flight altitude decreased.

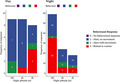

## 1 INTRODUCTION

Wildlife ecology and conservation has benefitted over the past decade from the emergence of drones, or unmanned aerial vehicles (UAVs), as a useful and innovative field research tool (Corcoran et al., 2021; Jiménez López & Mulero‐Pázmány, 2019; Linchant et al., 2015). Their relatively low cost and ease of use has seen them used for wildlife behavior, density and abundance monitoring (Chabot et al., 2015; Hodgson et al., 2013; Vermeulen et al., 2013), animal tracking (Cliff et al., 2018; Muller et al., 2019), antipoaching monitoring (Mulero‐Pázmány et al., 2014), recording of songbirds (Wilson et al., 2017), and the mitigation of human–wildlife conflict (Hahn et al., 2017). The potential of drones for data collection is only just beginning to be realized, but has already increased efficiency of processing and automation of data collection when compared to traditional ecological methods (e.g., ground truthing surveys on foot) (Hodgson et al., 2016; Martin et al., 2012).

Recent studies demonstrate that animal behavioral modification occurs as a result of drone surveying (Arona et al., 2018; Barnas et al., 2018; Bennitt et al., 2019; Brunton et al., 2019; Ditmer et al., 2015, 2019; Mulero‐Pázmány et al., 2017; Pomeroy et al., 2015). Missions that demand clear imagery, such as performing species counts and abundance mapping, require flying close to the animal without causing major movement or undesirable behaviors (e.g., birds mobbing the drone). The flight parameters (e.g., altitude, speed) that cause these disturbance behaviors are largely unknown, and therefore, the rules and guidelines designed to protect animals regarding drone flight are underdeveloped.

Detecting and accurately observing nocturnal species is a challenge that many researchers face and a key reason as to why nocturnal species are understudied compared to diurnal species (Vine et al., 2009). The cryptic nature and secretive movements of nocturnal animals, coupled with their adaptations to low light, make close observation extremely difficult (Balme et al., 2009; Jayasekara et al., 2007). Attempts have been made with drones to detect mammals during the night using thermal cameras (Chrétien et al., 2016; Seymour et al., 2017; Spaan et al., 2019; Zhang et al., 2020), with the primary objective to detect and count the target species. Evidence also exists that radio tracking of wildlife can be facilitated using drone systems (Cliff et al., 2015; Muller et al., 2019), reducing the labor and time costs of searching for animals in rugged terrain or thick vegetation and streamlining the process compared to traditional ground‐based tracking.

Currently, there is no information available as to whether there is a difference in the behavioral response exhibited by nocturnal animals to night‐time drone flight compared to daytime drone flight. It is to be expected that nocturnal animals display a different reaction as compared to diurnal animals to drone flight based upon the ability to identify the drone by the target species, coupled with the environmental conditions that influence the nature of the reaction (Bevan et al., 2018). The difference between sound propagation during the day and night is significant due to changes in temperature, humidity, wind speed, and atmospheric pressure within the atmospheric boundary layer (Embleton & Daigle, 1991). Increased turbulence during daylight hours and the formation of stable and reflecting layering of the lower atmosphere contribute to differences in sound propagation (Cosgrove, 1997). We hypothesize that these changes in environmental conditions have the potential to modify the behavior of species to drone flight from night‐time to daytime due to the difference in sound propagation properties.

The southern hairy‐nosed (SHN) wombat (*Lasiorhinus latifrons*), a nocturnal, burrowing marsupial herbivore (Taggart & Temple‐Smith, 2008), is a highly suitable model species to assess the behavioral response of nocturnal animals to drone flight. The large size of the wombat combined with its easily distinguishable shape against the harsh and barren landscape facilitates night‐time observations in their natural environment. Spotlighting is a common technique employed at night to observe their behavior from great distances, for which disturbance is minimal (Taggart et al., 2003). The home range of the SHN wombat is relatively small (1.3–4.8 ha) (Finlayson et al., 2005), and activity is undertaken close to their warrens which allows for straightforward location at night.

The aim of this study was to determine how drone flight activity influences the behavior of nocturnal animals. Here, we investigate the behavioral response of SHN wombats to drone flight at different altitudes during the day and night. Testing of this method is required to assess the validity of utilizing drones for automated collection of wombat behavioral information and for the remote tracking of SHN wombats. Bare‐nosed wombat warrens have previously been mapped successfully using a drone (Old et al., 2019), and therefore, it is important that the behavioral response of wombats to drone flight is explored to assess best practice flight methods. It is hypothesized that the animals will react more strongly to night‐time compared to daytime drone flight. Potential reasons are twofold; nocturnal animals have a more developed sense of hearing and night‐time meteorological conditions are more favorable for the propagation of sound.

## 2 MATERIALS AND METHODS

### 2.1 Study site

**FIGURE 1:**
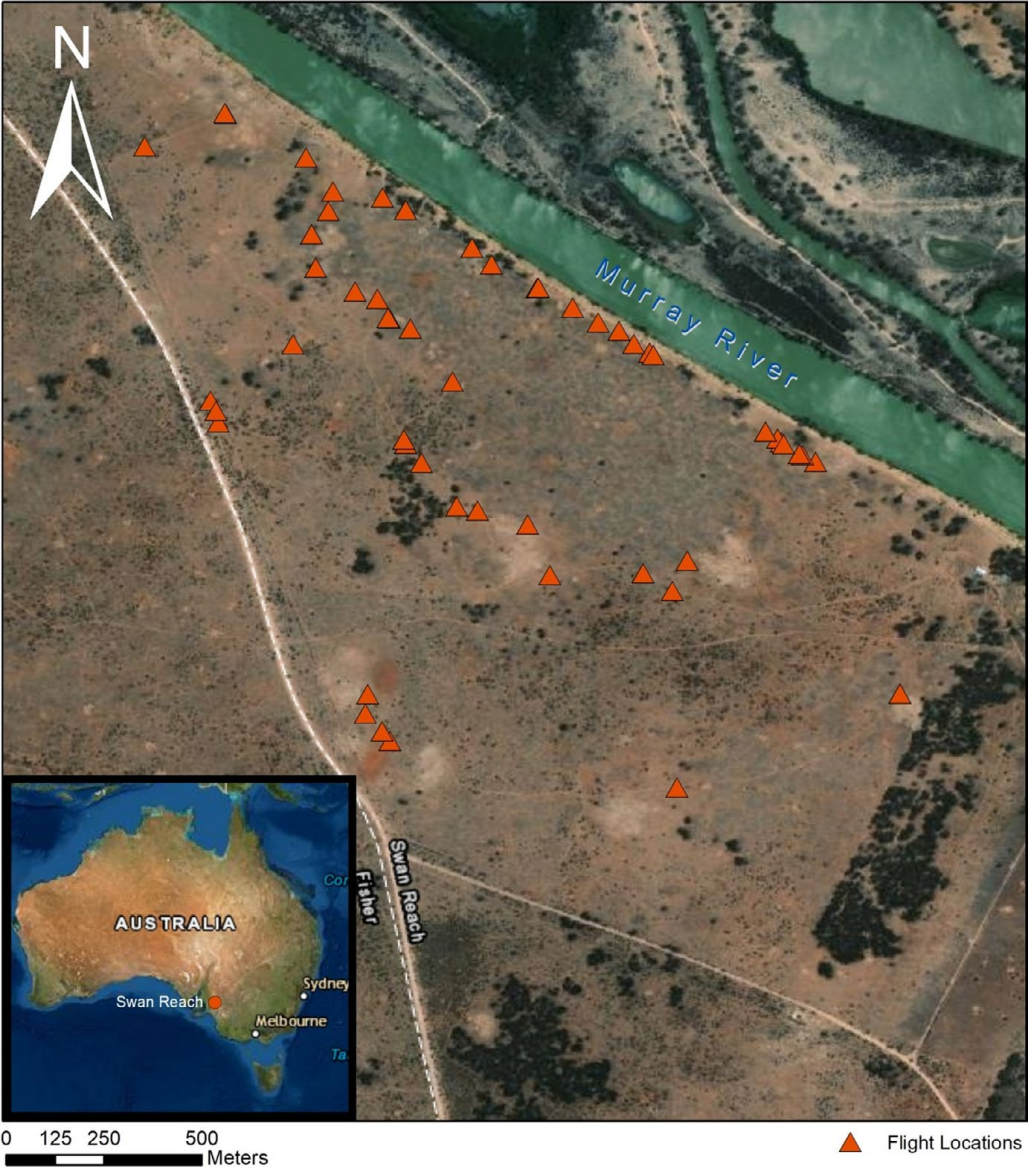
Locations of drone deployments on Kooloola Station, Swan Reach, South Australia. Drone deployments were undertaken on existing vehicle tracks on the property and located close to wombat warrens

This study was conducted at Kooloola Station, near Swan Reach (34°34′S 139°32′E), approximately 100 km north‐east of Adelaide in the Murraylands, South Australia (Figure 1). Southern hairy‐nosed wombats are found in high abundance on the station, with approximately 55,000 wombats estimated to live in the greater Murraylands region (Swinbourne et al., 2020). Three kangaroo species are also found on the property, including western kangaroos (*Macropus fuliginosus*), red kangaroos (*Macropus rufus*), and euros (*Macropus robustus*) (Taggart et al., 2020).

Kooloola Station is a farming property (sheep grazing) adjacent to the Murray River in among a semiarid mallee environment (*Eucalpyptus* spp.). The understory vegetation consists of saltbush (*Enchylaena, Atriplex*, and *Rhagodia* spp.) and bluebush (*Maireana* spp.) shrubs, with the herbaceous layer dominated by introduced weeds and small patches of native grasses (Taggart et al., 2007, 2020). Limestone (calcrete) forms the foundation for the soil, with zones of alluvial clay present (Walker et al., 2007). The area experiences a semiarid climate, characterized by hot, dry summers, (maximum 48°C) and cool dry winters (approximately 270 mm annual rainfall). The area is also periodically subjected to droughts (lowest 10% of rainfall records) (Taggart et al., 2003, 2020).

Kooloola Station has been commonly used for SHN wombat research across the past 25 years (Finlayson et al., 2005; Taggart et al., 1998, 2007, 2020), with regular monitoring activities including spotlighting, camera trapping, sample collection, and tagging/collaring. As a consequence of the high frequency of spotlighting undertaken at Kooloola, wombats have become habituated to the presence of human activity on nearby tracks and roads.

### 2.2 Drone flights

No regular drone flights have been conducted at Kooloola Station prior to this study; hence, the responses of the wombats to the drone were nonbiased due to the limited exposure.

#### 2.2.1 Test drone flights

Preliminary flights (*n* = 16 flights, *n* = 30 total transects) were flown over wombats using the same transect methodology and at the same flight altitudes used in the final data collection. The preliminary flights were conducted to refine the methodology and ensure reliable capture of data. The final data collection occurred during 3 consecutive days in October with similar weather conditions to reduce variability of responses.

#### 2.2.2 Drone flights and wombat behavior

A DJI Phantom 4 Pro™ drone was used during the final data collection. The drone flew horizontally at 5 m per second (m/s) directly over the located wombat/s at predetermined altitudes (100 m, 60 m, 30 m). After take‐off, the drone immediately ascended to an altitude of 100 m and flew along a transect over the wombat to a distance of approximately 100 m past the wombat and back to the launch position. If the wombat did not retreat to its burrow, the drone descended to the next lowest altitude (60 m) and the same horizontal transect was flown. This process was repeated until (a) the wombat retreated into its warren, or (b) all flight altitudes were exhausted. A single transect constituted flying over the wombat to the predetermined distance past the wombat (100 m) and back to the original launch position.

Sixty‐eight flights (*n* = 127 transects) were undertaken over wombats during daytime and night‐time, with 59 flights occurring during the night (*n* = 101 transects) and 9 taking place during day (*n* = 26 transects). Flights were on average 20 min apart during the day and approximately 14 min apart during the night. Despite some flights occurring a short distance apart from each other (*n* = 100 m), the temporal separation between flights was long enough for wombats to revert to their original behavior before location with the spotlight.

##### Locating wombats for daytime drone flights (5:30 p.m.–7:30 p.m.)

Searching for wombats for daytime drone flight experiments involved driving a 4WD vehicle slowly along tracks on the property searching for wombats on, or near, their warrens. Once a wombat was spotted, the vehicle was positioned behind the closest bush and stopped to ensure the wombat was not disturbed prior to launching the drone. The drone was then deployed at a safe distance from the vehicle and out of sight of the wombat. The observers stood motionless and semi‐obscured from the wombat to avoid any disturbance and monitored the animal's behavior through binoculars (8 × 40 mm).

##### Locating wombats for night‐time flights (9:30 p.m.–2 a.m.)

Searching for wombats at night involved two observers walking along the vehicular tracks at the station using a spotlight (Ledlenser™ H14R.2 headlamp; Low 60 lumens, Power 450 lumens and Boost 1,000 lumens) to scan for wombats on or near their warrens. The drone pilots followed behind at a distance of approximately 100 m. If a wombat was identified, a red light was used to signal to the drone pilots to stop and set up ready to launch the drone. While this was happening, the spotlight was shone on the wombat for ~2 min to ensure that any change in behavior observed following deployment of the drone was caused by the presence of the drone and the sound it created and not associated with the spotlight. Previous experience spotlighting wombats over multiple surveys across decades has indicated that wombats are highly tolerant of spotlights, of which poor eyesight can be attributed to. Typically no change is observed in behavior following detection by spotlight unless the animal is within a close proximity (~25 m).

#### 2.2.3 Field data collection and behavioral observations

Detailed notes and observations were made associated with each drone flight. Information was collected on time, flight altitude, location, moon phase, latitude, and longitude of drone launch site on the property, Warren ID, distance between the drone launch site and the wombat (Yukon™ Extend LRS‐1000 Rangefinder) and wombat behavior.

Wombat behavior was classified as follows:

No behavioral response exhibited (0)
Alert but stationary (1)
Alert with movement in any direction, but did not retreat to burrow (2)
Alert and retreat into burrow (3)

SHN wombat behavior was categorized and tabulated into an ethogram (Table 1). Behavioral observations using binoculars (8 × 40 mm) occurred approximately 40–120 m from the wombat/s during drone flights. Other anthropogenic sources of disturbance, such as cars driving on a road adjacent to the property, or planes flying overhead, were noted but were rarely present. In the event these disturbances occurred, flights were postponed until vehicles were clear of the area and the study environment returned to its natural state. A wombat was considered alert if it was observed to lift its head and prick up its ears as a result of the disturbance activity (e.g., drone noise, spotlight, vehicle noise). If there was no change in behavior pre‐ and post‐drone flight, the wombat was considered to have been unaffected behaviorally by drone flight.

**TABLE 1:** Classification of SHN wombat behavior to night‐time and daytime drone flight

Type of behavior	Description of behavior
No Response (0)	No observable reaction is displayed. Animal maintained original behavior (e.g., resting and feeding)
Alert, no movement (1)	Focal animal is alert, head raised, and ears pricked up, searching for the source of the sound
Alert with movement, but no retreat into burrow (2)	Focal animal is alert, head raised, and ears pricked up, searching for the source of the sound; and displacement of animal from original position
Alert and retreat into burrow (3)	Focal animal is alert, head raised, and ears pricked up, searching for the source of the sound, full retreat into burrow

### 2.3 Data analysis

All statistical analyses of wombat behavior taken from the field behavioral observations were undertaken in the R environment (R Core Team, 2020). A general linear mixed effects model (GLMM) was used to evaluate the significance of the covariates time of day, flight altitude, moon phase, and distance between launch site and the wombat. None of these covariates except flight altitude had a significant effect on wombat behavior.

#### 2.3.1 Survival analysis

In order to evaluate the response of wombats to drone flight altitude, we used the "survival" package (Therneau, 2020). In this instance, "survival" denotes a wombat remaining above ground and not retreating into its burrow. "Survival" probability was calculated as a response to drone altitude, but not for time. This analogy allows for the estimation of confidence intervals around the wombats staying above ground and to separate between night and day responses.

### 2.4 Ethics

This study was conducted under the University of Adelaide Animal Ethics permit number S‐2018‐112a. All drone flights were undertaken with prior approval from the Civil Aviation Safety Authority (CASA), and the pilot was in the possession of a Remote Pilot Licence (RePL).

## 3 RESULTS

**FIGURE 2:**
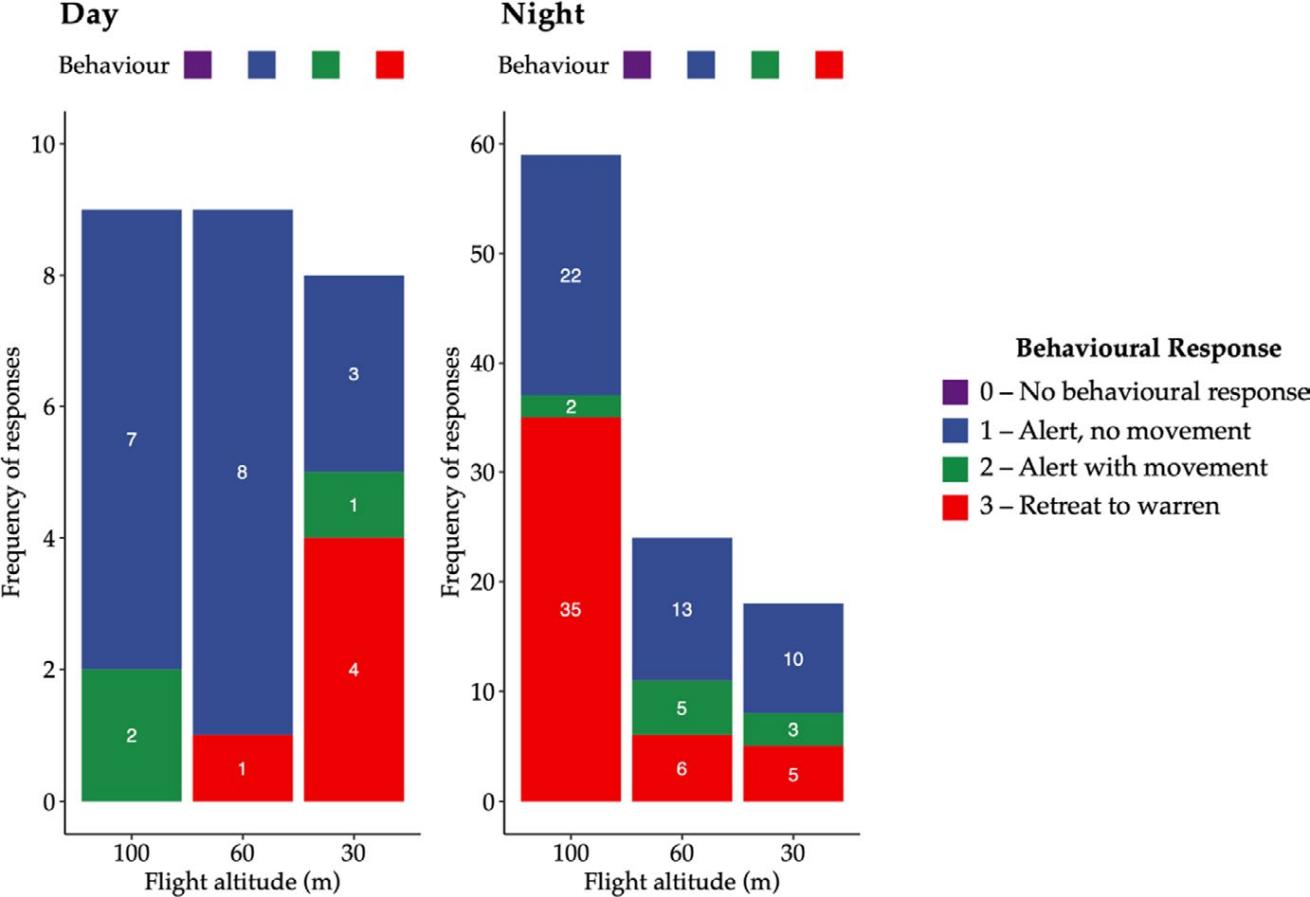
The count of each class of southern hairy‐nosed wombat behavioral responses to drone flight, at different flight altitudes and across both daytime (left) and night‐time (right) flights

Behavioral responses differed depending upon drone flight altitude and time of day (Figure 2). We were able to locate 9 wombats during daylight hours and 59 animals during the night. All wombats exhibited a form of vigilance behavior (1, 2, and 3) during flights, and no wombats were observed to be undisturbed by the drone irrespective of time of day. Note that there is no behavior response type 0 at any altitude in Figure 2.

Stationary alertness was the most commonly observed behavior during the day at 100 and 60 m altitudes. At night, only 22 of 59 wombats exhibited this behavior. The type "Alert with movement" was the least frequent behavior. If a wombat was displaced from its original position, it was also likely to retreat.

Across all flight altitude classes, wombats were observed to increase their frequency of retreat in response to drone flight altitude descent. Of the 9 observed wombats, 5 retreated and 4 remained above ground. This contrasts to 46 of 59 night‐time retreats and only 13 remaining (Figure 2). A total of 35 wombats retreated at 100 m flight altitude during night‐time flights, and a further 6 and 5 retreated at 60 m and 30 m altitude respectively for a total of 46 wombat retreats. Five wombats retreated during daytime flights; one at 60 m altitude and a further four at 30 m altitude. The highest frequency of wombat retreats occurred for night‐time flights at the highest flight altitude (100 m; *n* = 35), while during the day this was observed at the lowest flight altitude (30 m; *n* = 4).

**FIGURE 3:**
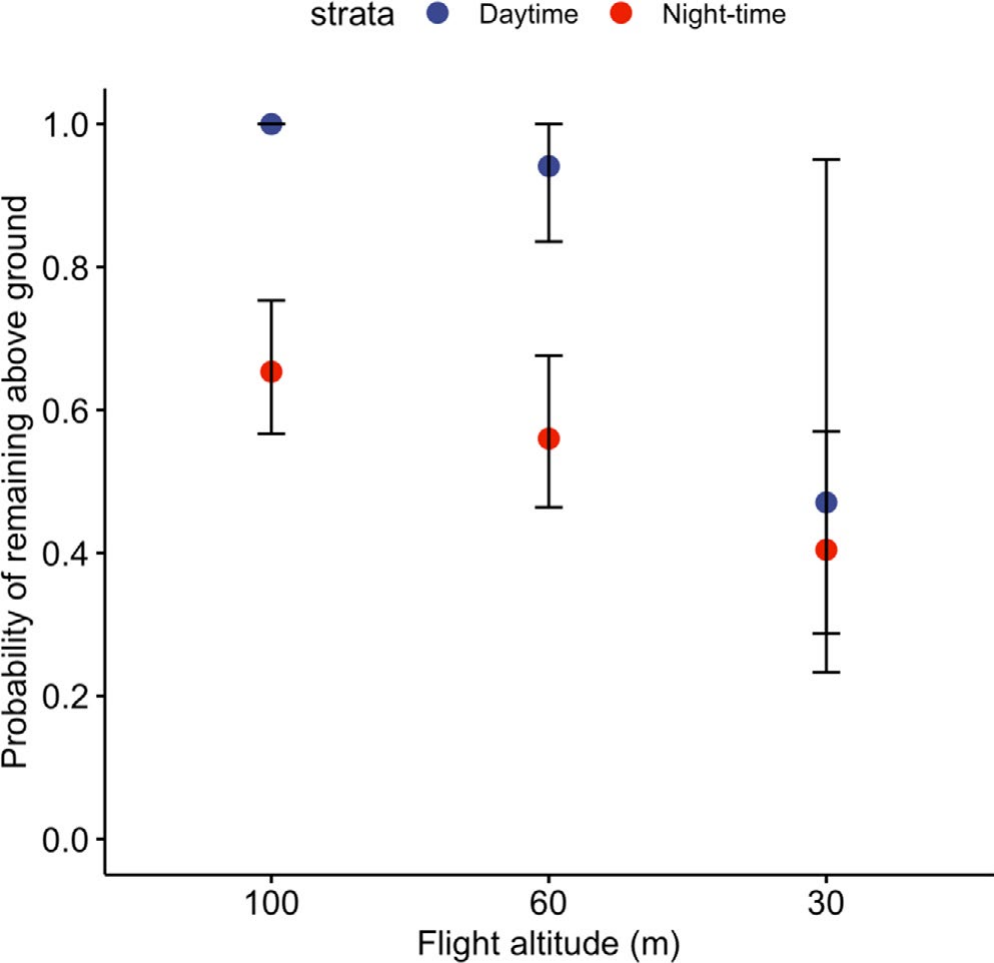
Probability of a southern hairy‐nosed wombat remaining above ground as a function of flight altitude and time of day. The error bars represent the 95% confidence interval

The probability of a wombat remaining above ground as a function of flight altitude and time of day varied considerably (Figure 3). A statistically significant difference was observed between the "survival" probability of wombats at drone flight altitude 100 m (Day 100%; (*n* = 9); conf int. 1–1, Night 65%; (*n* = 59); conf int. 0.75–0.56) and 60 m (Day 94%; (*n* = 9); conf int. 1–0.83, Night 56%; (*n* = 24); conf int. 0.67–0.46). However, no significant difference was observed between "survival" probability for daytime (47%; *n* = 8; conf int 0.95–0.23) or night‐time flights (40%, *n* = 18; 0.56–0.28) when the drone was flown at 30 m altitude.

## 4 DISCUSSION

This study is the first to examine the disturbance behavior of a nocturnal mammal to drone flight with a comparison between night‐time and daytime behavioral responses. It also establishes the southern hairy‐nosed wombat as an excellent model species for examining the behavioral responses of mammals to drone flight.

The results demonstrate that the presence of the drone elicited a behavioral response for all wombats across all flight altitude classes, regardless of time of day.

These results are consistent with observations undertaken on other animals. A study conducted on 7 African herbivore species, of which the drone was either lowered vertically toward the animal from >100 m or approached horizontally at either 10, 20, or 30 m above ground level from approximately 400 m away, showed that the type of response and the tolerance of the drone varied for each species (Bennitt et al., 2019). Some wombats displayed a higher tolerance level to the drone by remaining above ground after the drone had flown over at 30 m altitude, although this was an uncommon occurrence relative to retreat behavior (*n* = 17). The results suggest that flight altitude is a significant factor of instigating disturbance behaviors for not only daytime but night‐time drone flight. Currently, little is known on the disturbance behaviors of any nocturnal animal to drone flight; however, it is likely that the different responses of the wombats to drone flight at night compared to daylight hours was influenced by its sensory adaptations, ability to detect the drone, the sound emitted by the drone, its state of fear and the environmental conditions at the time of drone flight (Bevan et al., 2018).

It is difficult to quantify the extent to which wombats were stressed during drone flight, although behavioral movements indicate that the presence of the drone and the noise emanating from it caused disturbance behaviors. Common behavioral changes in animals due to noise include the modification of movement patterns, a decrease in foraging behavior and increased vigilance and anti‐predator behavior (Shannon et al., 2016). During drone flight, wombat movement patterns were altered with animals becoming more vigilant and with a significant increase in retreat to warren behavior. This would suggest that time spent foraging would be significantly reduced and vigilance/anti‐predator behavior significantly increased due to drone presence. These behavioral alterations can influence physiological stress levels and individual survival, negatively impacting fitness (Fardell et al., 2020; Francis & Barber, 2013). Infrequent drone flights are, however, very unlikely to cause any long‐lasting behavioral and stress‐related impacts on an animal. Regular and repeated drone flights may elicit a stronger response in an animal or may result in the habituation of the animal to the presence of the drone and the sound it emits (Ditmer et al., 2019), resulting in a reduction in their perceived threat of the drone and behavioral response. This area of study requires further investigation.

Night‐time meteorological conditions at the field site during drone flights differed markedly to daytime conditions, with time of day appearing to influence the ability of the wombat to detect the drone. During night‐time flights, wombats commonly appeared to be searching for the source of the sound, and this was characterized by the lifting of the wombat's head and the pricking up of its ears following drone deployment. These observations confirm that wombats could detect the drone auditorily. It is likely that the wombats were able to hear the drone well before they could see it due to their reputably poor eyesight (Triggs, 2009) associated with their nocturnal and burrowing lifestyle. This lifestyle would favor a heightened sense of smell and hearing, rather than keen eyesight (Taggart & Temple‐Smith, 2008). In this study, the drone was flown at heights potentially too high for the wombats to detect the red and green lights on the drone. A large percentage of the wombats observed at night instantly pricked up their ears as soon as the rotors of the drone started spinning for take‐off, and less so during the day, most likely due to the scattering loss of sound and masking of drone sound from wind and atmospheric turbulence (Attenborough, 2007; Barber et al., 2010). This observation is consistent with a study of African herbivores, for which the animals also appeared to search for the sound of the drone when it was well out of eyesight (Bennitt et al., 2019). Awareness of the sensory capabilities of the target species is important for the implementation of appropriate study design that allows for minimal disturbance.

Sound propagation varied significantly with time of day. During this study, night‐time conditions were mostly clear with low wind (low cloud cover, average wind speed <5 km/hr). These conditions give rise to ground‐based temperature inversions (Attenborough, 2007; Wilson et al., 2015), causing strong downward refraction of sound. As sound levels decline at a slower rate during inversions, this allows sound to travel faster and more directly between two points in these conditions, and in this case, rapidly and more directly downward from the drone to the wombat. Daytime conditions at the field site were also relatively clear with low wind (low cloud cover, wind speed <5 km/hr). High solar radiation conditions result in higher atmospheric turbulence with unstable stratification and upward refraction of sound (Embleton & Daigle, 1991), rendering it more difficult for the wombat to hear the sound emitted from the drone flying overhead. It is likely that the change in conditions from daytime to night‐time altered the sound propagation properties emitted from the drone and hence altered the sound characteristics and associated behavioral reaction of the wombats studied.

It is highly probable that the noise emitted from the drone is a novel anthropogenic sound for the wombats, given that the location of the study site is away from any significant urban centers. Such sounds have the potential to cause disturbance behaviors dependent upon the perceived predation risk by the target species (Meillère et al., 2015; Quinn et al., 2006; Shannon et al., 2014). SHN wombats inhabiting the study site do not fall victim to any form of animal predation, either on land or from above. The shape and size of the drone when flying may resemble several aerial predators in the region, such as Whistling Kites (*Haliastur sphenurus*), Black Kites (*Milvus migrans*), and Wedge‐tailed Eagles (*Aquila audax*), but wombats do not appear worried by their presence. However, the noise of the drone appeared to disturb the wombat, suggesting that the wombat perceived the sound as a threat. Anthropogenic noise has been documented to increase vigilance behaviors of animals, and, if the threat is deemed severe enough, cause fleeing of the area (Ware et al., 2015). This was the response of some wombats to the drone and the noise it emitted. It is also common for some species to show no behavioral response to potentially fearful stimuli but elicit physiological symptoms. This has been demonstrated in a study on black bears and their response to drone flight (Ditmer et al., 2015) and is likely the case for the "stationary alert" wombats that did not retreat. Factors such as age, sex, reproductive status, and body condition score all influence an animals perception of predation risk (Gaynor et al., 2019), and it is likely that these factors influenced the vigilance behavior observed in some form.

## 5 CONCLUSION

The ability of drones to collect data efficiently and with high precision and accuracy is changing the way field researchers design ecological studies. Despite drone use becoming more common in the field, little is known about how animals react to drone flight, particularly at night. The marked difference in behavioral response between day and night flights observed in this study suggests that drone flight has a different influence on the behavior of nocturnal animals compared to previous studies on diurnal species (Bennitt et al., 2019; Bevan et al., 2018). Studies of nocturnal animals that involve drone flight should acknowledge this difference. It is likely that the strong behavioral differences, observed between day and night flights can be reduced by habituation. Test flights to observe and gauge the reaction of the target species prior to primary drone field tests may therefore be appropriate.

## CONFLICT OF INTEREST

None declared.

## AUTHOR CONTRIBUTIONS

**Taylor Headland:** Conceptualization (equal); formal analysis (lead); investigation (lead); project administration (equal); software (equal); writing–original draft (lead); writing–review and editing (lead). **Bertram Ostendorf:** Conceptualization (equal); formal analysis (equal); funding acquisition (lead); investigation (equal); methodology (equal); project administration (equal); resources (equal); supervision (lead); validation (equal); writing–review and editing (lead). **David Taggart:** Conceptualization (lead); formal analysis (equal); investigation (equal); methodology (lead); project administration (equal); resources (equal); supervision (equal); validation (equal); writing–review and editing (equal).

## Data Availability

Data used to undertake analyses are available at Dryad (https://doi.org/10.5061/dryad.5dv41ns6c).
